# Rydberg positronium injection of electrons and positrons into a levitated dipole trap

**DOI:** 10.1140/epjd/s10053-026-01191-2

**Published:** 2026-08-17

**Authors:** A. Deller

**Affiliations:** Max-Planck-Institute für Plasmaphysik, 85748 Garching, Germany

## Abstract

**Abstract:**

A neutral-beam injection scheme for magnetically trapping electrons and positrons is proposed. Pulsed lasers excite positronium (Ps) to long-lived Rydberg states that drift at speeds of $|\vec {v}| \sim 10^5$ $\textrm{m} \, \textrm{s}^{-1}$ into the magnetic field of a levitated superconducting coil. The electric field experienced in the reference frame of the rapidly moving atoms is sufficient to ionize a significant portion of the loosely bound Ps. The released electrons and positrons are confined to the poloidal magnetic field lines that wrap around the coil. Rydberg positronium injection (RPI) does not perturb the confinement volume and ensures local charge symmetry. The technique provides equal, low temperatures for the electron and positron distributions, as well as smooth, inwardly peaked density profiles. The range of populated field lines depends on the Ps velocity distribution and the ionization threshold of the Rydberg state. Monte Carlo simulations are used to evaluate the $e^+$ efficiency of RPI for the APEX (A Positron–Electron eXperiment) levitated dipole.

**Graphical abstract:**

**Figure:**
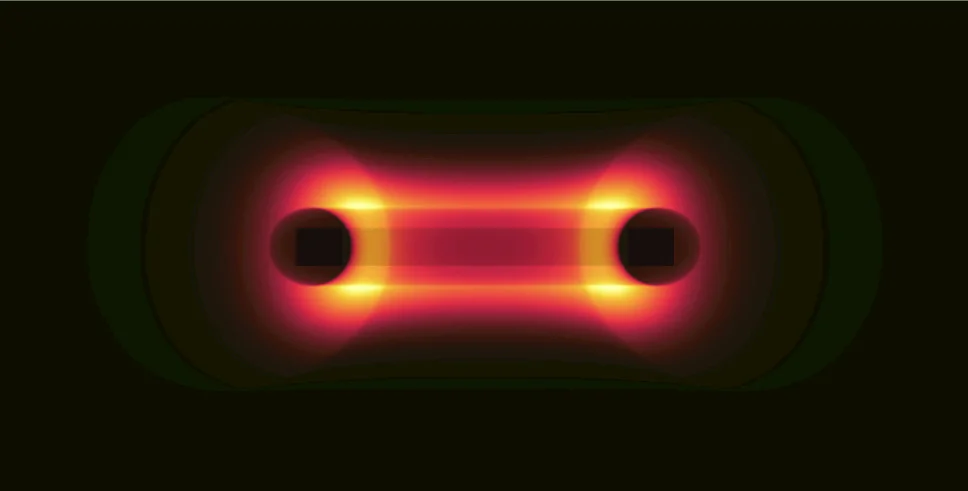

## Introduction

Magnetized plasmas of ions and electrons are prone to instabilities and turbulent dynamics that are infamously difficult to predict or control. An important example is anomalous turbulent transport: a highly nonlinear, multiscale effect that commonly limits confinement in magnetic fusion devices [1]. Pair plasmas are a special class of quasi-neutral plasma composed of positive and negative particles with the same mass. Every constituent particle, therefore, responds to perturbations on similar time scales, and many of the mechanisms that seed instabilities in magnetized ion-electron plasmas are expected to be absent or suppressed [2]. Relativistic electron–positron plasmas likely occur in various extreme astrophysical environments [3]. Collections of MeV pairs at plasma densities can also be generated in the laboratory using powerful pulsed lasers [4, 5] or GeV proton beams [6]. In the low-temperature regime ($k_BT \sim 1$ eV), stable magnetic confinement is conceivable [7], which could facilitate unprecedented investigations into instabilities and transport in magnetized plasmas with perfect mass symmetry. Accordingly, there has been a great deal of progress toward the co-confinement of low-energy electrons and positrons at—or approaching—plasma densities [8–11]. However, trapping of a pair plasma, with a Debye length, $\lambda _D$, that is much shorter than the size of the collection [12], has not yet been accomplished.

The APEX (A Positron–Electron eXperiment) project aims to create, confine, and study low-temperature pair plasmas in the magnetic field of a levitated current ring [13]. Construction of the high-temperature superconducting levitating dipole (LD) was recently completed [14, 15]. Initial tests have demonstrated long levitation times (>3 hours) and stable confinement of pure electron plasmas (>10 s) [16]. Whereas a linear trap requires electrostatically biased end-cap electrodes [17], the closed magnetic field lines of APEX-LD can be used to simultaneously confine electrons and positrons in the same volume. The simple coil geometry allows for a relatively compact confinement volume that minimizes the number of positrons needed to create a neutral pair plasma. Filling the trap will nonetheless require record quantities of cold (eV) positrons ($N \gtrsim 10^{10}$ $e^+$) [13], which must be transferred from an accumulation device into the closed confinement volume.

Modern positron trapping techniques [18–22] can be used to accumulate a non-neutral plasma that contains $N \sim 10^9$ $e^+$ in a single Penning–Malmberg trap [23, 24]. The maximum number of $e^+$ that can be practically accrued is limited by the strength of slow-$e^+$ sources [25] and the amount of space charge that can be held in a linear trap ($\textrm{kV}$) over long accumulation times (hours). Multi-cell traps [26–28] are being developed in response to the limited capacity of conventional Penning–Malmberg traps. In combination with upgraded low-energy positron sources [29–31], accumulation of $N > 10^{10}$ $e^+$ is expected with the next generation of trapping devices [32].

Drift injection is currently the most promising method for transferring non-neutral positron plasmas into APEX-LD. This technique uses an electric field to instigate an $\vec {E} \times \vec {B}$ drift of the charged particles across the transport magnetic field lines and onto the closed field lines of the dipole [33]. Simulations and experiments with supported dipoles have progressively refined this technique [34, 35], and an injection efficiency of 100% was recently demonstrated using a bunched positron beam [36]. However, drift injection necessarily perturbs the trapping volume. Accordingly, to achieve good confinement, the electric field must be removed immediately after injection [36, 37]. Cooling can be exploited to stack consecutive bunches [20]; however, this has not yet been demonstrated in a purely magnetic trap. Although drift injection can probably be adapted to work with large non-neutral plasmas, it will be challenging to merge $e^-$ and $e^+$ plasmas with significant space-charge potentials without considerable heating.

Recently, there have been major advances in the experimental techniques for producing and controlling the electron–positron bound state, positronium (Ps) [38]. This quasi-stable atom has a total spin of either S=0 (para-Ps) or S=1 (ortho-Ps). In vacuum, the ground state (principal quantum number n=1) of the hydrogen-like system has an average lifetime against self-annihilation of 125 ps for para-Ps and 142 ns for ortho-Ps [39]. Positronium formation is, therefore, a potentially important loss channel for a cold electron–positron plasma. Nevertheless, in the density and temperature regimes anticipated for APEX-LD, the radiative and three-body recombination rates are expected to be ≪1 Hz [13].

Rydberg states of positronium (n≳10) are essentially stable against annihilation [40]. A beam of laser-excited $\text {Ps}^*$ can, therefore, be sufficiently long-lived to move equal numbers of electrons and positrons from an external source into a magnetic trap. The motional Stark effect (MSE) provides a mechanism to ionize the rapidly moving, weakly bound Ps atoms in the regions of the device with the strongest magnetic fields [41]. Rydberg positronium injection (RPI) solves several of the problems associated with charged-particle injection. The concept is a form of neutral-beam injection (NBI), which is widely employed in magnetic fusion devices [42]. In 1986, *Surko et al.* proposed using a pulsed beam of ground-state ortho-Ps to deposit positrons into a tokamak plasma for transport studies [43]. This idea prompted the development of the buffer-gas positron trap [18], which went on to have an extraordinary impact on the field of antimatter physics [22]. Although the original diagnostic was never implemented, field ionization of excited states of Ps has been employed to accumulate positrons in ultra-high-vacuum Penning traps [44, 45]. Elements of RPI are shared with schemes for using Rydberg Ps to produce low-energy antihydrogen atoms via charge-exchange with antiprotons [46–49]. Laser excitation of specific Rydberg states can be very efficient [50] and provides exquisite control over the charge-exchange reaction [51]. The concept is attractive for low-energy pair plasma studies [7] because it can transport equal quantities of electrons and positrons into strong magnetic fields with negligible heating. The principal quantum number of the Rydberg atoms can be optimized according to the magnetic field geometry and desired density distribution. The technique is compatible with pulse stacking and ensures the oppositely charged electrons and positrons occupy the same magnetic field lines. However, for all its inherent advantages, RPI is inevitably much more expensive—in terms of positrons and hardware—than lossless drift injection [34].

**Fig. 1:**
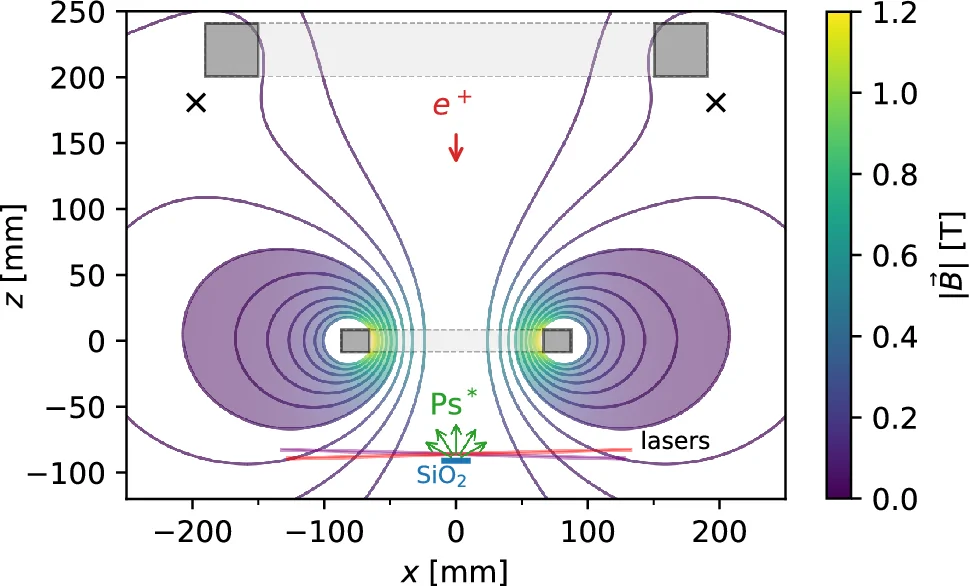
The RPI scheme for APEX-LD. A bunch of positrons is implanted into mesoporous silica to produce Ps atoms that are excited by pulsed lasers to Rydberg levels. The solid lines represent magnetic field lines. The line color indicates the magnitude of the magnetic flux density. The upper and lower gray boxes show the positions of the L- and F-coil. The ×-points mark where the fields of the two coils sum to zero. The shaded region indicates the poloidal cross-section of the plasma confinement volume

This article evaluates the practicality of implementing RPI with APEX-LD. The injection concept and relevant experimental details are described in Sect. 2. Results of trajectory simulations and calculations of the Rydberg level that maximizes the injection efficiency are presented in Sect. 3. Then in Sect. 4, important considerations not included in the simulations are discussed. Finally, the feasibility of using RPI to trap enough electrons and positrons to create a pair plasma is reviewed.

## APEX-LD

### Overview

APEX-LD utilizes a high-temperature superconducting (HTS) floating (F-)coil and a water-cooled copper lifting (L-)coil [15] (see Fig. 1). The F-coil is levitated inside a 45-cm-diameter cylindrical vacuum chamber on top of which the 34-cm-diameter L-coil is concentrically affixed. The F-coil has a radius of $R_\textrm{F} = 75$ mm and is wound from 150 turns of 12-mm-wide rare-earth barium copper oxide (ReBCO) [52] HTS tape. To prepare the F-coil for levitation, it is cooled to 20 K and then inductively energized with a persistent current of $I_F \sim 60$ kA-turns. The 1.3 kg F-coil is then magnetically levitated by supplying $I_L \sim 2.9$ kA-turns to the lifting coil. The vertical position of the F-coil is measured by laser displacement sensors and stabilized by a proportional-integral-derivative (PID) feedback circuit that controls the L-coil supply current. Although the persistent current flowing in the HTS tape steadily decays, the PID controller can maintain the position of the F-coil at the center of the vacuum chamber for over 3 h [14]. The fully energized F-coil generates an on-axis (r=0) magnetic flux density of $B_0 \sim 500$ mT. Quasi-neutral or non-neutral plasma can be magnetically confined within the toroidal volume bounded by the poloidal field lines that wrap around the levitating coil [53, 54]. The maximum possible size of this volume is constrained by the last closed field line, which intersects the magnetic null indicated by the ×-points in Fig. 1. However, the APEX-LD vacuum chamber limits the usable confinement region to a smaller volume of V∼10 liters; the plasma trapping volume is indicated by the shaded area in the same figure.

### Injection scheme

The RPI scheme is shown in Fig. 1. A non-neutral plasma of positrons is accelerated to ∼2 keV and time-focused to $\Delta t \sim 5$ ns [55]. The magnetic field of the LD guides the positrons along the axis of symmetry of both coils and into a mesoporous silica target. Embedded $e^+$ thermalize in the bulk silica and form Ps atoms that enter the interconnected network of 5-nm pores with average kinetic energies of $\langle K \rangle \sim 1$ eV [56]. Inelastic collisions with the walls of the pores rapidly cool the Ps as they diffuse toward the surface of the film [57]. A substantial fraction of the initial bunch is re-emitted to vacuum as positronium, with a conversion efficiency of $\epsilon _\textrm{Ps} \sim 0.4 / e^{+}$ [58]. Loss channels include direct annihilation, para-Ps formation, and pick-off annihilation of ortho-Ps inside the pores [59]. Epithermal ortho-Ps atoms are emitted from the mesoporous silica with typical speeds of $|\vec {v}|\sim 1.7 \times 10^5$ $\textrm{m} \, \textrm{s}^{-1}$ [60]. In vacuum, the triplet ground-state lifetime only allows for a mean flight path of a few cm. For the Ps atoms to reach the LD, they must be excited to Rydberg levels. These have long lifetimes and can be populated using ultraviolet (UV), λ=243 nm, and infrared (IR), $\lambda \sim 740$ nm, ns-pulsed lasers that drive electric-dipole transitions from the ground state ($1S \rightarrow 2P \rightarrow n S/D$) [50, 61].

Rydberg positronium is more likely to radiatively decay than to self-annihilate in the excited state [62]. The mixed-ℓ fluorescence lifetime (ℓ is the orbital angular momentum quantum number) is $\tau _f \approx 10$ μs for n=14 and increases with $n^4$ [40]. Laser-excited $\text {Ps}^*$ may therefore drift across the magnetic field lines until they reach the chamber walls (∼1 μs) or the MSE ionizes them [41]. If ionization occurs within the confinement volume, the unbound electrons and positrons will become trapped. The gradient and curvature of the magnetic field will result in a slow toroidal drift around the floating coil [63], with opposing drift directions for the newly separated positive and negative charges.

**Fig. 2:**
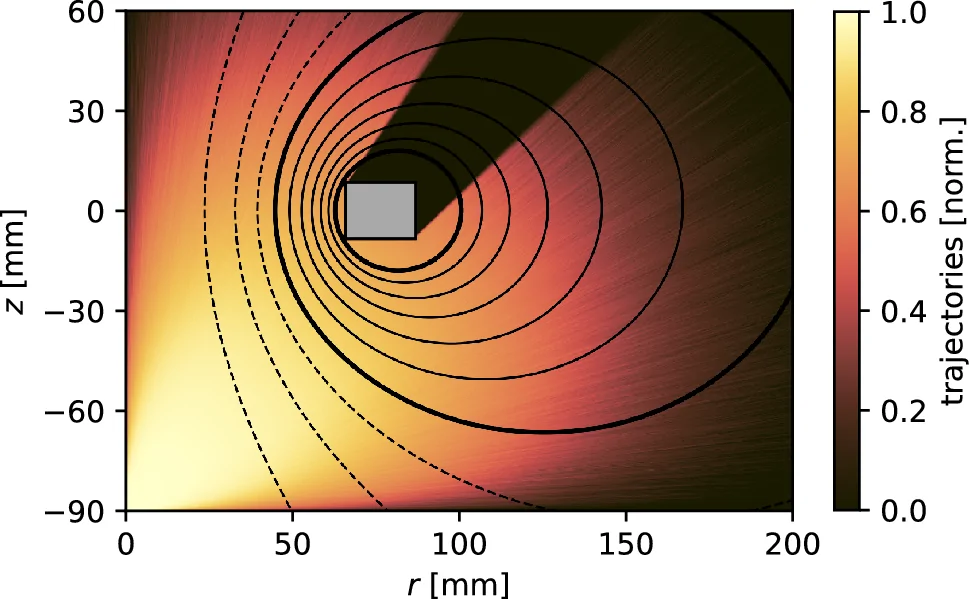
Trajectories of $N = 10^6$ Rydberg Ps projected on the *rz* plane (t<600 ns). The equalized color scale indicates the number of overlapping paths. The Ps source is located at z=-90 mm. The gray box shows the position of the F-coil. The solid [dashed] lines represent magnetic field lines inside [outside] the confinement volume (bounded by thick, solid lines)

## Simulations

In this section, three-dimensional Monte Carlo (MC) simulations are used to evaluate the overlap between Rydberg positronium trajectories and the confinement volume of APEX-LD. The MSE along the Ps flight paths is calculated to determine the ionization locations for different Rydberg states.

### Trajectories

The simulated Ps atoms originate at the mesoporous silica converter, which is located beneath the F-coil at z=-90 mm. Each atom is assigned a velocity, $\vec {v}_{i}$, which is determined by random sampling from a T=1500 K thermal speed distribution, a cosine distribution of emission angles with respect to the vertical (*z*) axis [64], and a uniform distribution of azimuthal angles. All of the emitted Ps atoms are assumed to be immediately excited to Rydberg levels with a fluorescence lifetime that is much longer than the flight time. Sampling of the phase space distribution by the finite size and bandwidth of the pulsed lasers is neglected. As discussed in Sect. 4.1, velocity selection by the lasers is important; however, the objective of this calculation is to determine the acceptance of the entire distribution. No forces act on the simulated atoms and they travel in straight lines away from the mesoporous silica, i.e., $x_i(t) = x_i(0) + \vec {v}_i t$.^1^ Figure 2 shows the first 600 ns of $N = 10^6$ MC simulated trajectories projected onto the *rz* plane. The toroidal confinement volume is defined by the 2π-revolution about the dipole axis (r=0) of the area between the poloidal field lines with equatorial (z=0) crossings at $r_0 = 100.7$ mm and 206.5 mm; approximately 80% of the trajectories intersect this volume.

**Fig. 3:**
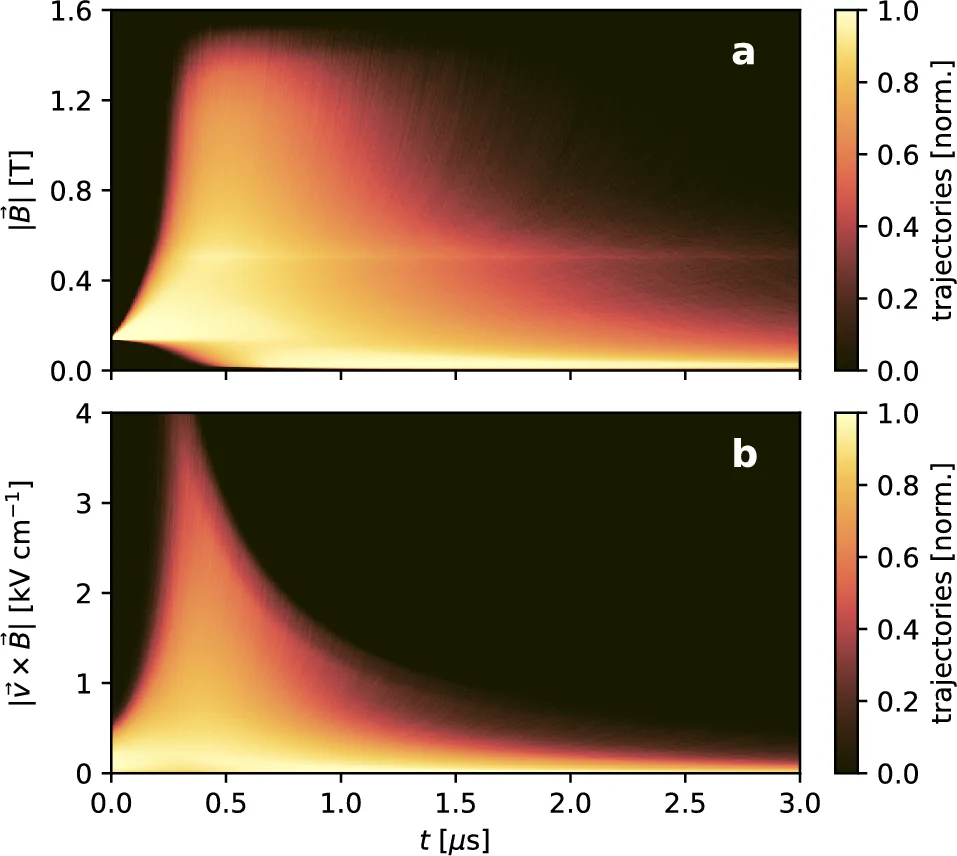
The amplitude of the **a** magnetic and **b** electric field experienced along $10^6$ Rydberg Ps trajectories. The equalized color scales illustrates the number of overlapping lines (arb. units)

**Fig. 4:**
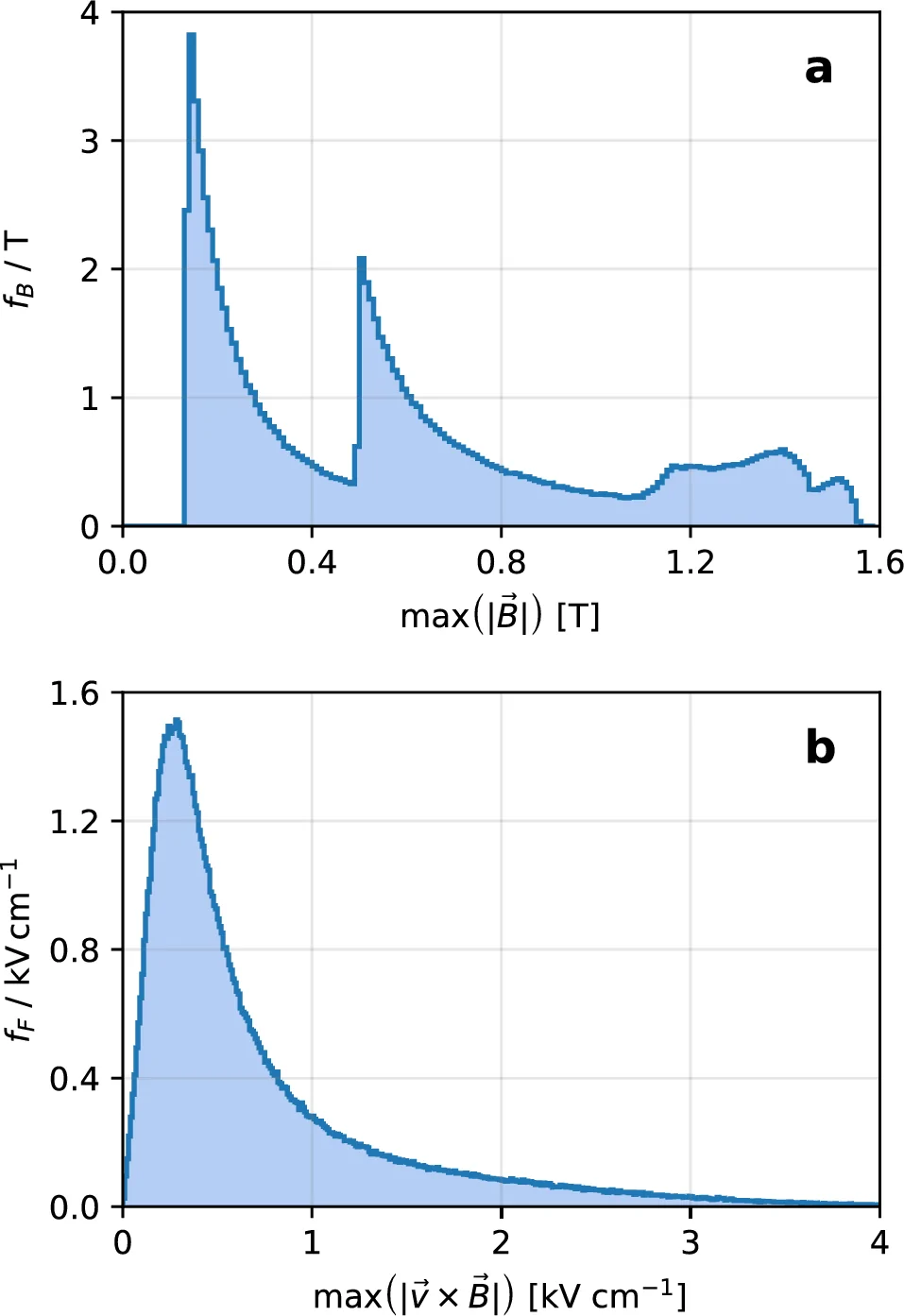
Density distributions, $f_X$, of the maximum value along each trajectory of **a** $|\vec {B}(x_i)|$ and **b** $|\vec {v}_i\times \vec {B} (x_i)|$, where $x_i(t)$: $i \in \left\{ 1,2, \dots , N\right\}$ represents time-dependent coordinates in 3D Cartesian space for an MC simulation of $N = 10^6$ Rydberg Ps atoms

The magnitude of the magnetic field, $B = |\vec {B}|$, experienced along each trajectory is shown in Fig. 3a. In the strongly inhomogeneous field, the Ps distribution experiences a broad range of field amplitudes. Trajectories that pass through the center of the F-coil experience magnetic field strengths of at least B=0.5 T. The atoms that fly directly toward the HTS winding pack encounter fields greater than 1 T shortly before colliding with it. The weakest fields are experienced by those that pass under the coil. The range of maximum magnetic field strengths experienced by the distribution of trajectories is presented in Fig. 4a.

Positronium has a low mass ($M = 2 m_e$), and it tends to move at high speed. Accordingly, most of the trajectories reach the equatorial plane of the F-coil in under 1 μs. Because the co-rotating electron and positron have a net circulating current of zero, Zeeman shifts are unusually small [66–68]. Nevertheless, Ps atoms moving rapidly across a large magnetic field will experience a significant Lorentz electric field [69],

1 $$\begin{aligned} \vec {E} = \vec {v} \times \vec {B} \, . \end{aligned}$$

Figure 3b shows the strength of the motional electric field, $F = |\vec {E}|$, experienced along each flight path; the largest fields exceed F=4 $\textrm{kV} \, \textrm{cm}^{-1}$. At t∼400 ns, the fastest Ps atoms enter the region containing the strongest magnetic fields. The distribution of the maximum electric fields experienced along the trajectories is shown in Fig. 4b. This is smoother than the histogram of magnetic field strengths shown in Fig. 4a because the motional electric field depends on the direction of the magnetic field as well as its amplitude. Although many of the trajectories pass through regions of high magnetic field strength, only those that fly toward the winding pack encounter strong fields that are orthogonal to their direction of travel.

### Ionization

**Fig. 5:**
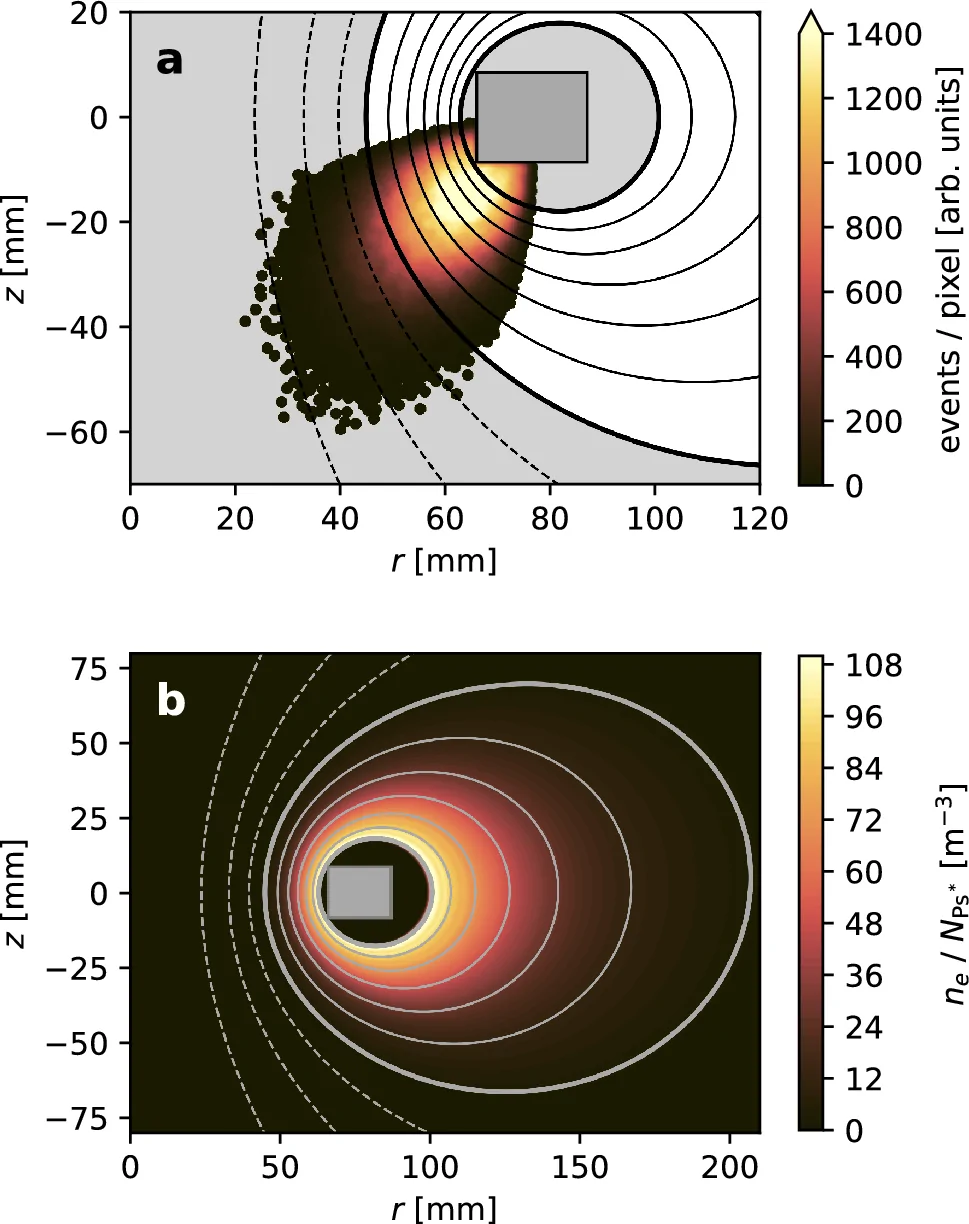
**a** Locations in the *rz* plane of 175,788 field ionization events from $N_\mathrm {Ps^*} = 10^6$ calculated trajectories with an ionization threshold of $F_\textrm{ion} = 1.2$ $\textrm{kV} \, \textrm{cm}^{-1}$. The color scale indicates the number of overlapping points. **b** The subsequent trapped lepton density, assuming the released electrons and positrons become uniformly distributed within flux tubes. The gray box shows the position of the F-coil, and the lines represent magnetic field lines. Dashed field lines are outside the confinement volume

Rydberg atoms are sensitive to electric fields [70], and the weakly bound excited states are easily field-ionized [41, 71]. Figure 5a shows the *rz* coordinates at which the trajectories presented in Fig. 2 first encounter a Lorentz electric field in excess of $F_\textrm{ion} = 1.2$ $\textrm{kV} \, \textrm{cm}^{-1}$. These locations are here classified as ionization events; 17.6% of the calculated trajectories experience a field above the threshold. They are predominantly associated with trajectories that fly toward the floating coil with an emission angle close to $40^\circ$ from the vertical axis ($\hat{z}$). 75.1% of the ionization events occur inside the confinement volume (13.2% of all Rydberg Ps trajectories). RPI predominantly injects electrons and positrons into the strong magnetic field region of the LD trap. The released leptons are strongly magnetized and tightly bound to the magnetic field lines on which ionization occurs. Ionization products from events that occur before the $\text {Ps}^*$ enter the confinement volume follow the magnetic field lines into the vacuum chamber wall. Similarly, ionization in the region shadowed by the winding pack does not contribute to the captured population.

Each of the closed poloidal field lines of the confinement volume can be uniquely labeled according to the radial coordinate of its outboard equatorial crossing, $r_0$. The outermost field line, $r_0 = 206.5$ mm, encloses a toroidal volume of V=0.0138 $\textrm{m}^{3}$. The magnetic field strength along this field line varies from $B_\textrm{min} = 13.6$ mT to $B_\textrm{max} = 693$ mT with a mirror ratio $B_\textrm{max} / B_\textrm{min} = 51$. By contrast, the innermost field line, $r_0 = 100.7$ mm, is limited by the F-coil case; it encloses a toroidal volume of V=0.00055 $\textrm{m}^{3}$ and has a mirror ratio of $B_\textrm{max} / B_\textrm{min} = 4.7$.

Captured particles with small pitch angles (between the velocity vector and the magnetic field), $\alpha _\theta = \arctan (v_\perp / v_\parallel )$, travel along each field line with a constant parallel speed, $v_\parallel \approx |\vec {v}|$. If this applied to the entire population, the density would vary within each flux tube in proportion to the magnetic flux density, which strongly peaks in the inboard region. However, a charged particle with a non-negligible perpendicular speed, $v_\perp$, moving adiabatically along a magnetic field line, will continuously exchange parallel and perpendicular energy to conserve its magnetic moment $\mu = m_e v_{\perp }^2 / 2 B$; this effectively repels particles from the high-field regions of the trap. Collisions will lead to an increasingly isotropic velocity distribution and a population of mirror-trapped particles on the outboard side of the levitating F-coil [72]. Figure 5b illustrates the total lepton density in the *rz* plane, assuming the injected particles experience no radial transport after ionization and that they approach a uniform distribution along magnetic field lines.^2^

Transport across the field lines of the cold, diffuse collection of strongly magnetized particles will be slow compared to thermalization along the field lines [74]. However, assuming negligible volumetric losses (i.e., recombination and annihilation), radial transport, due to collisions, asymmetries, or instabilities, will nevertheless eventually redistribute the ionization products [75–77].

### Rydberg level optimization

Like hydrogen, positronium can be modeled as a single electron with a reduced mass, $\mu _e = m_e / 2$, orbiting a fixed positive charge [78]. The Ps atom, therefore, has a hydrogenic energy level structure (excluding fine structure and higher-order corrections [79, 80]) but with roughly half the binding energy of H,

2 $$\begin{aligned} E_n = -\mu _e c^2 \alpha ^2 \frac{1}{2 n^2} \approx -\frac{6.8}{n^2} \, [\textrm{eV}], \end{aligned}$$

where *c* is the speed of light in vacuum and $\alpha \approx 1 / 137$ is the fine-structure constant.

**Fig. 6:**
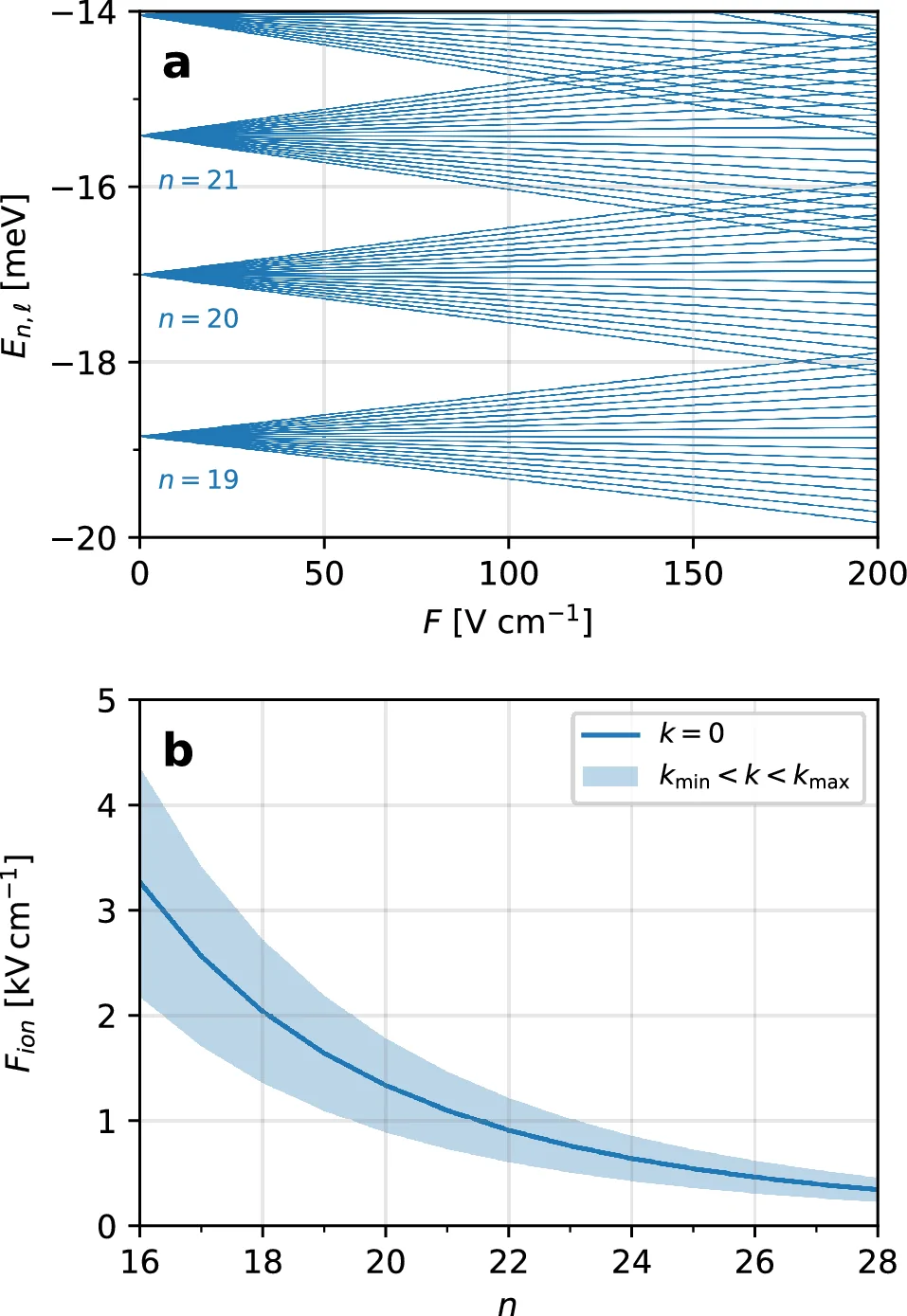
**a** Stark-shifted energy levels of n=19, 20 & 21 Rydberg Ps (m=2) in a weak electric field. **b** The range of ionization electric fields, $F_\textrm{ion} = [F_\textrm{class}, 2 F_\textrm{class}]$, for the n=16 to 28 Rydberg–Stark states of Ps

Figure 6a shows the Stark splitting of n=19 to n=21 Rydberg positronium (for magnetic quantum number m=2) in an electric field of magnitude, $F = |\vec {E}|$. The electric field breaks the spherical symmetry of the Hamiltonian and splits the ℓ-degenerate energy levels into the Rydberg–Stark manifold [81]. In a weak electric field, the Stark shift depends linearly on the electric field strength [82],

3 $$\begin{aligned} \Delta E_{n, k} = \frac{3}{2} n k e a_\textrm{Ps} F \, , \end{aligned}$$

where $a_\textrm{Ps} = \hbar / \mu _e c \alpha \approx 0.1058$ nm is the Bohr radius of positronium, ħ is the reduced Planck constant, and $k = n_1 - n_2$ (where $n_1$ and $n_2$ are quantum numbers in parabolic coordinates [83]), which can take integer values in the range of ±(n-|m|-1) in increments of Δk=2. The Stark shifts are either positive (k>0) or negative (k<0), depending on how the charge distribution is aligned with the applied electric field.

The electric field can be increased until the saddle point of the total electric potential is low enough for the orbital electron to escape. This provides the classical limit for field ionization [71, 82],

4 $$\begin{aligned} F_\textrm{class} = \frac{\mu _e c^2 \alpha ^2}{e a_\textrm{Ps}}\frac{1}{9 n^4} \approx \frac{1.4284 \times 10^5}{n^4} \, [\mathrm {kV \, cm^{-1}}] , \end{aligned}$$

which applies to the outermost state with a negative Stark shift (k≈-n). Tunneling through the barrier is also possible [84, 85]. However, the ionization rate decreases exponentially for fields below the classical limit. Field magnitudes of approximately $2 F_\textrm{class}$ are needed to ionize the most positively Stark-shifted states (k≈n) [86]. Figure 6b shows the range of ionization fields for the Rydberg Ps levels n=16 to 28; the solid line represents the mean value, $F_\textrm{ion} = 1.5 F_\textrm{class}$, which is approximately equal to the electric field amplitude required to ionize the k=0 state [87].

**Fig. 7:**
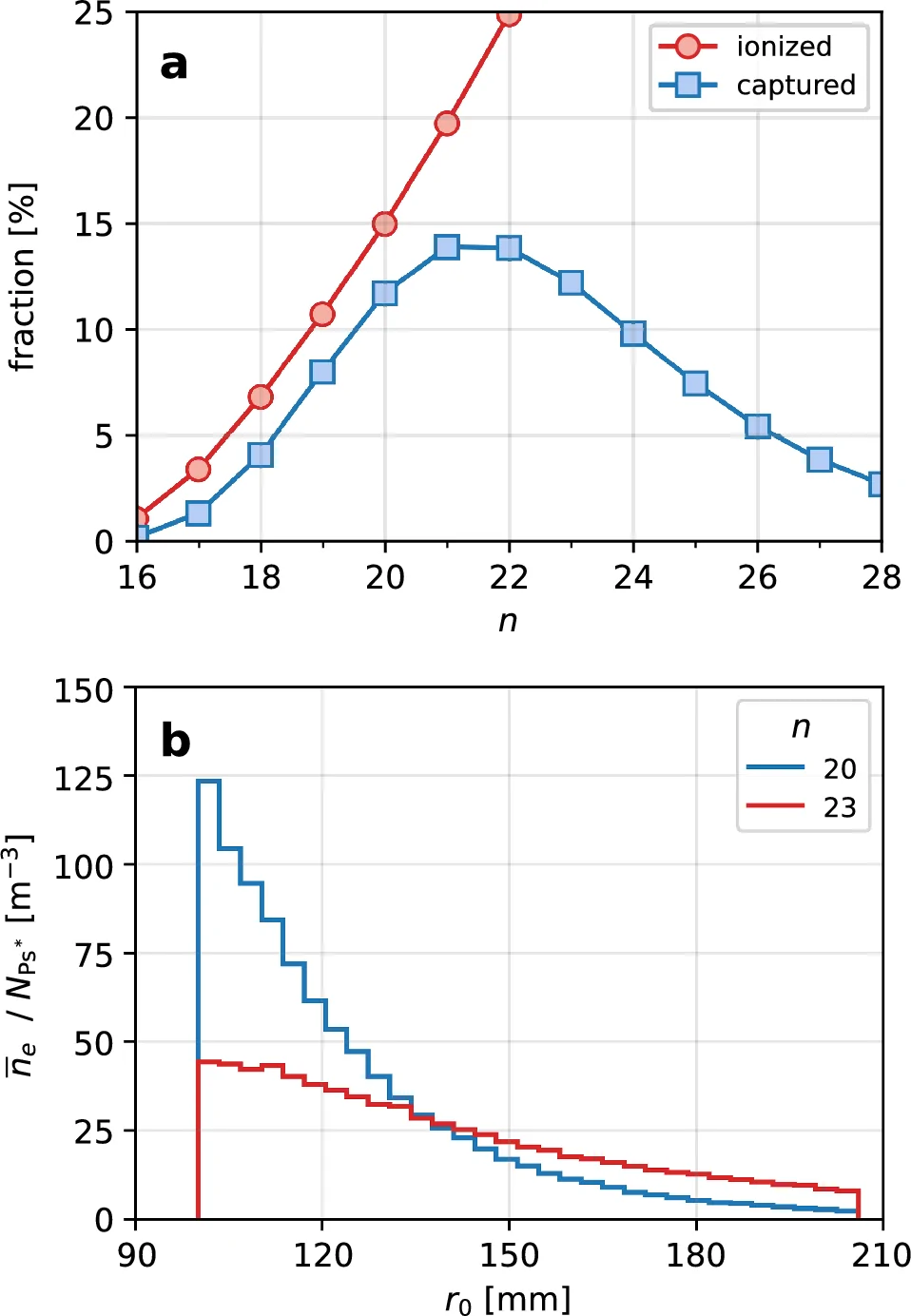
**a** The fraction of $N_\mathrm {Ps^*} = 10^6$ simulated Rydberg Ps atoms that are field-ionized (circles) and subsequently captured (squares) for n=16 to n=28 and k=0. **b** Flux-tube-averaged captured lepton density per Rydberg Ps atom for n=20 and 23

Figure 7a shows the fraction of calculated Rydberg Ps trajectories that are ionized due to the motional Stark effect for different values of *n*. The field ionization threshold was set to $F_\textrm{ion} = 1.5 F_\textrm{class}$. The ionized fraction is ∼1% for n=16 and ∼65% (off scale) for n=28. The fraction of the distribution that ionizes inside of the confinement volume initially tracks the ionized fraction before peaking at $\epsilon _\textrm{cap} = 13.9$% for n=21. States with higher values of *n* ionize more easily; however, progressively fewer electrons and positrons are captured because the ionization threshold is increasingly met in weaker fields outside the confinement volume.

**Fig. 8:**
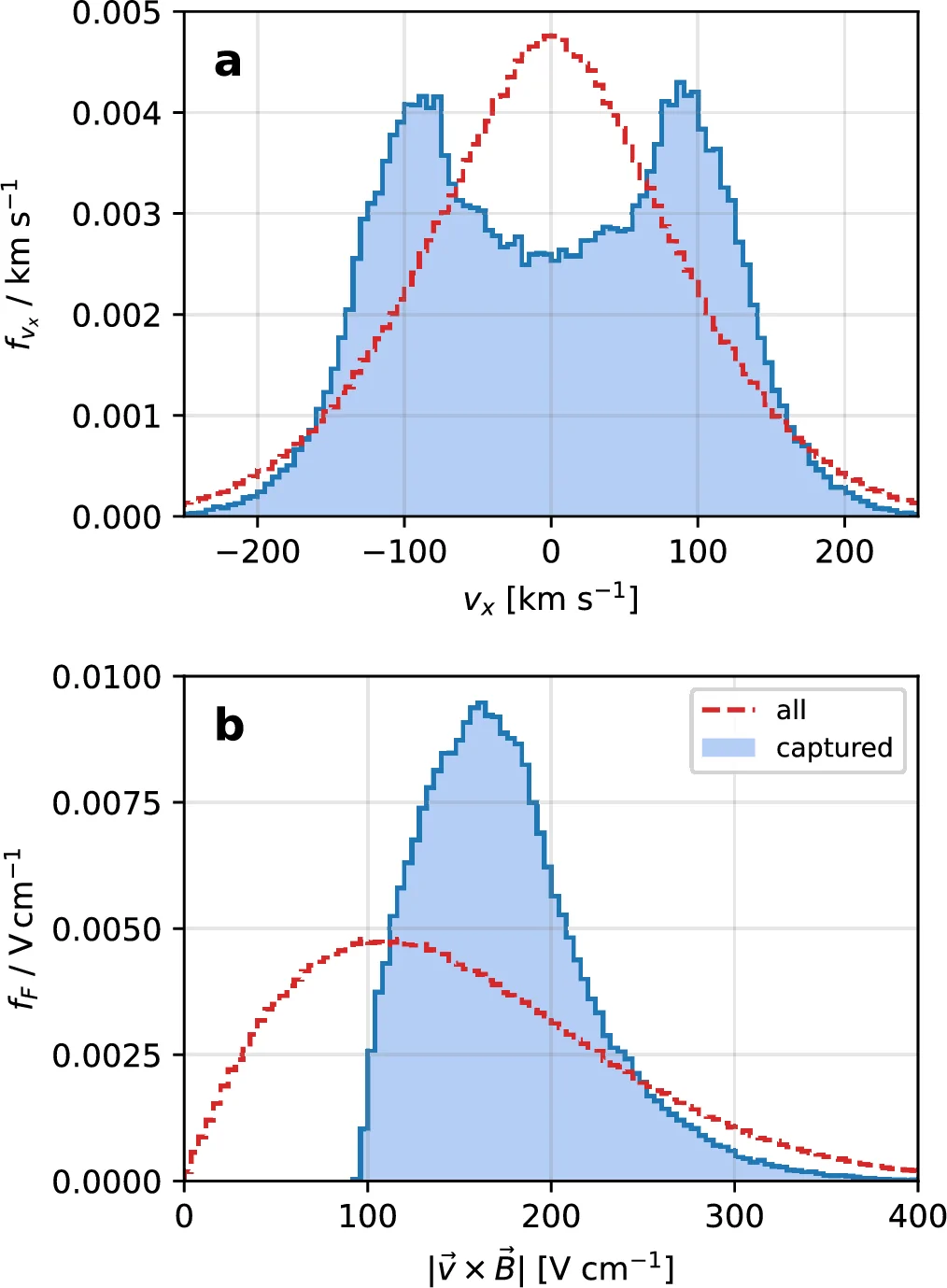
Captured Rydberg Ps distributions for an ionization threshold of $F_\textrm{ion} = 1.2$ $\textrm{kV} \, \textrm{cm}^{-1}$ (shaded). **a** The *x*-component of the velocity. **b** The initial Lorentz electric field experienced at r=0, z=-90 mm ($B_z = 134$ mT). The dashed lines represent the equivalent distributions for all $N_\mathrm {Ps^*} = 10^6$ trajectories

The different ionization thresholds of the Rydberg levels offer experimental control over the ionization profile by tuning the wavelength of the infrared laser [81]. In general, lower-*n* states penetrate further into the trap and ionize nearer the floating coil. Targeting the region close to the floating coil is beneficial for obtaining higher densities and lower temperatures of the trapped charged particles (see Sect. 4.4 for further discussion). Figure 7b provides a comparison of the density profile of the $e^-$ and $e^+$ ionization products for Rydberg Ps levels n=20 and n=23 and k=0. The results verify that a much more inwardly peaked profile can be obtained with the lower Rydberg state. The calculated capture efficiencies for n=20 and n=23 were $\epsilon _\textrm{cap} = 11.7$% and $\epsilon _\textrm{cap} =12.2$%, respectively. Although the maximum capture efficiency, $\epsilon _\textrm{cap} = 13.9$%, was found for n=21 ($F_\textrm{ion} \approx 1.1$ $\textrm{kV} \, \textrm{cm}^{-1}$), the highest peak density was obtained with n=19 ($F_\textrm{ion} \approx 1.6$ $\textrm{kV} \, \textrm{cm}^{-1}$).

### Injection efficiency

The total positron efficiency for RPI into APEX-LD can be estimated using,

5 $$\begin{aligned} \epsilon _\textrm{inject} = \epsilon _\textrm{Ps} \times \epsilon _\textrm{ex} \times \epsilon _\textrm{cap} \, . \end{aligned}$$

The total efficiency is $\epsilon _\textrm{inject} \approx 1.4$%, assuming: $\epsilon _\textrm{Ps} = 40$% of the $e^+$ implanted into the mesoporous silica are emitted to vacuum as ortho-Ps [58]; $\epsilon _\textrm{ex} = 25$% of the ground-state Ps can be laser-excited to Rydberg levels [50]; and $\epsilon _\textrm{cap} = 14$% of the $\text {Ps}^*$ are field-ionized by the MSE as they traverse the strong magnetic field of the LD confinement volume. Note that this definition of efficiency only takes into account losses incurred during injection (positrons that are lost during moderation or accumulation are not included).

## Discussion

In this section, several important effects that are relevant to RPI but are not included in the simulations are discussed. The feasibility of using RPI to create an electron–positron plasma with presently available $e^+$ trapping technology is also assessed.

### Doppler broadening

The Doppler broadening of the optical transitions used to excite the distribution of Ps atoms can be extremely large. The Lyman-α transition (1S→2P) has a typical Doppler width of $\Delta f_\textrm{FWHM} \sim 1$ THz [88]. This compares to a bandwidth of ∼100 GHz for the λ=243 nm UV laser that is usually employed to drive the transition [57, 89]. Although pulsed lasers can provide sufficient fluence to saturate and power broaden this transition, Doppler broadening is nonetheless a major component of $\epsilon _\textrm{ex}$ [50].

The trajectory calculations reveal a significant correlation between the *x* component of the velocity distribution (which is associated with the Doppler shift of the laser) and the perpendicular velocity that determines the Lorentz electric field that ionizes the excited atoms. Figure 8a shows the distribution of $v_x$ for the captured trajectories, assuming an ionization threshold of $F_\textrm{ion} = 1.2$ $\textrm{kV} \, \textrm{cm}^{-1}$, compared to the full distribution. The velocities along the *x* direction for the entire population are normally distributed with a peak at $v_x = 0$, whereas the captured trajectories have a double-peak distribution with maxima at $v_x = \pm 84$ $\text {km/s}$. The implicit assumption of Eq. 5, that $\epsilon _\textrm{ex}$ and $\epsilon _\textrm{cap}$ compound independently, is therefore overly pessimistic. The maximum capture efficiency would be obtained by detuning and retro-reflecting the UV laser to address the subset of the Ps distribution that is most likely to be ionized in the confinement region of the levitating dipole.

### Stark broadening

The range of motional Stark shifts experienced by the distribution of Ps atoms in the laser excitation region is expected to be the main contributor to the width of the 2P→nS/D transition. Along the axis of symmetry of the coils, the magnetic field is vertical: $\vec {B}(r=0) = B_z \hat{z}$, with $B_z \approx 134$ mT at z=-90 mm. Figure 8b shows the t=0 electric field distribution for the calculated trajectories that were later field-ionized inside of the confinement volume for an ionization threshold of $F_\textrm{ion} = 1.2$ $\textrm{kV} \, \textrm{cm}^{-1}$. Because the captured trajectories have a relatively narrow range of emission angles, they are also associated with a notably narrower spread of initial Lorentz electric fields than the full Rydberg Ps distribution.

For the Rydberg levels of interest, the Lorentz electric fields at the excitation region are comparable to the Inglis–Teller limit [90], beyond which adjacent Rydberg–Stark manifolds overlap—see Fig. 6a. For n=19, an electric field amplitude of F=150 $\textrm{V} \, \textrm{cm}^{-1}$ is just below this limit, but it would nevertheless result in significant ℓ-mixing and splitting of the Rydberg–Stark manifold. Similar splittings have been exploited to populate specific Rydberg–Stark states [81]. However, as shown in Fig. 8b, the Ps atoms experience a range of electric fields that reflect the spread of perpendicular speeds. Although the associated broadening would inhibit the selection of states with specific values of *k*, it should be possible to resolve predominantly k>0, k≈0, or k<0 states [65]. If so, lower Rydberg levels could be utilized. As shown in Fig. 6b, the outermost negatively Stark-shifted state of the n=19 Rydberg level has a ionization field of $F_\textrm{ion} \approx 1.1$ $\textrm{kV} \, \textrm{cm}^{-1}$, which is roughly equal to $F_\textrm{ion}$ for the k=0 state of the n=21 level.

Motional Stark broadening would be lessened by moving the excitation region further from the floating coil. However, this would also reduce the geometric acceptance of the LD. Fortunately, Stark broadening does not necessarily preclude efficient laser excitation of positronium to high-*n* states. *Cassidy et al.* [50, 62] demonstrated efficient $1S \rightarrow 2P \rightarrow n S/D$ laser excitation of Ps atoms to Rydberg levels despite sizable magnetic, $|\vec {B}| = 160$ mT, and electric, $|\vec {E}| = 200$ $\textrm{V} \, \textrm{cm}^{-1}$, fields in the laser-interaction region. The excitation efficiency, $\epsilon _\textrm{ex} \sim 25$%, was mainly limited by the frequency overlap between the UV laser and the Doppler-broadened Lyman-α transition. The fraction of the Ps atoms that were excited to Rydberg levels was considerably higher than might be expected for a stochastically populated 3-level system [67], which was attributed to suppressed stimulated emission from the upper level due to the intermixing of the Rydberg–Stark states in the presence of the fields. Distinct Rydberg levels up to n≈15 were observed as well as overlapping manifolds up to n≈25. *Wall et al.* [81] performed similar measurements in a much weaker magnetic field and resolved individual Rydberg Ps levels up to n≈30.

**Fig. 9:**
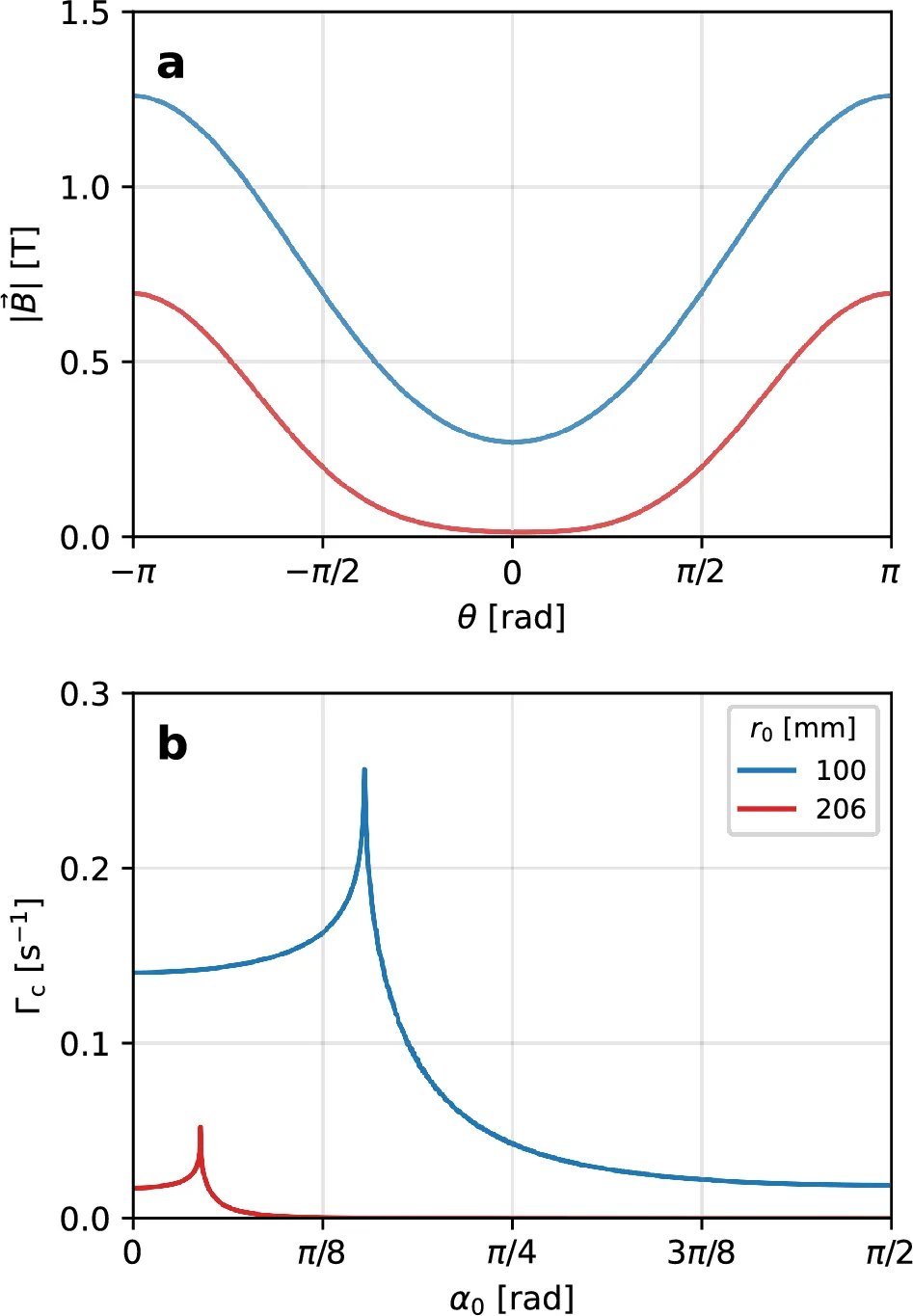
**a** The magnitude of the magnetic flux density along the inner and outer poloidal field lines of the confinement volume [key in (**b**)]. **b** The dependence on the equatorial (θ=0) pitch angle, $\alpha _0$, of the bounce-averaged cyclotron cooling rate for the inner and outermost field lines

### Cyclotron cooling

The Monte Carlo distribution used in Sect. 3 has an average kinetic energy of $\frac{3}{2} k_B T = 194$ meV. The mean kinetic energy of the captured subset of trajectories is $\langle K \rangle = 245$ meV for an ionization threshold of $F_\textrm{ion} = 1.2$ $\textrm{kV} \, \textrm{cm}^{-1}$. Whereas the initial kinetic energy of the full distribution is equally divided between parallel and perpendicular motion, the captured trajectories are ionized with $\langle K_\parallel \rangle = 33$ meV and $\langle K_\perp \rangle = 213$ meV. The semi-classical mechanics of the ionization of cold Rydberg atoms in a magnetic field is a complicated topic [91–94]. Nevertheless, it is reasonable to assume that the electrons and positrons will separate with an equal share of the kinetic energy of the ionized atoms, i.e., ∼100 meV.

The confinement time of a pure electron plasma in an LD trap can exceed 100 s [54]. Magnetized electron–positron plasmas are expected to be remarkably stable [2], even in the curved magnetic fields of the dipole trap (for small density and temperature gradients) [77]. Furthermore, losses due to recombination and annihilation are expected to be slow [13]. It is therefore conceivable that the Ps ionization products will be confined long enough to cool via the emission of cyclotron radiation [95].

The electron cyclotron cooling rate is given by [21, 96],

6 $$\begin{aligned} \Gamma _c = \frac{2 e^{4} B^2}{9 \pi \varepsilon _0 m_e^3 c^3} \approx 0.26 B^2 \, \left[ \textrm{s}^{-1} \right] , \end{aligned}$$

with *B* in units of Tesla ($\varepsilon _0$ is the vacuum permittivity). Due to the inhomogeneity of the dipole magnetic field, the cyclotron cooling rate varies significantly throughout the confinement volume. Figure 9a shows the magnetic field strength along the inner and outer field lines (θ represents the poloidal angle with θ=0 for coordinates on the outboard equatorial plane of the F-coil). The cooling time can be estimated from the line-average of $B^2$, which gives $1 / \Gamma _c = 7$ s for $r_0 = 100.7$ mm and $1 / \Gamma _c = 58$ s for $r_0 = 206.5$ mm. This assumes that the charged particles equally sample all segments of each field line, which only applies to those with equatorial pitch angles, $\alpha _0$, that are almost zero. As shown in Fig. 9b, the bounce-averaged cooling rate for particles with large pitch angles is much lower because magnetic mirroring traps them in the low-field region. This calculation uses the weighted average of $B^2$, with line-segment weights of $1 / v_\parallel$ (assuming μ is adiabatically conserved along each field line). The peaks of enhanced cooling correspond to small but nonzero pitch angles with turning points inside the high-field region of the trap.

### Pair plasma

For the captured electron and positron distributions to qualify as a pair plasma, the Debye length,

7 $$\begin{aligned} \lambda _D = \sqrt{\frac{\varepsilon _0 k_B T_e}{n_e e^2}} \, , \end{aligned}$$

should be shorter than the size of the collection [12] (here, $n_e = n_- + n_+$ is the *total* lepton number density). In this regime, the charged particles shield externally applied electric fields, and collective effects can arise [97]. The shortest length scale of the APEX-LD confinement volume is the inboard span of field lines, L∼1 cm.

**Fig. 10:**
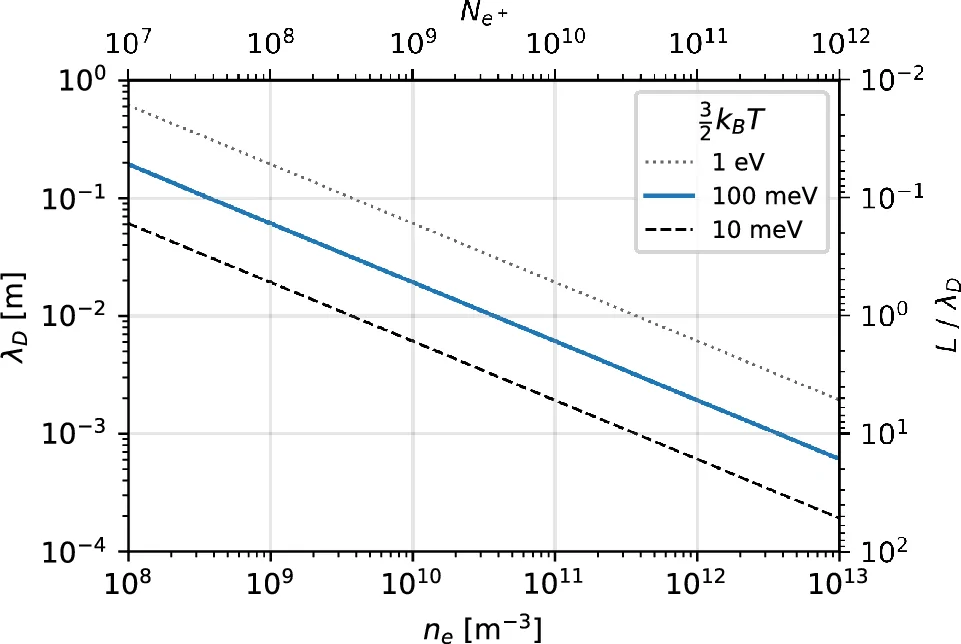
Debye length dependence on the total lepton density for a given plasma temperature (key inset). The top axis represents the estimated number of positrons required to achieve a certain density using RPI, including the estimated Ps production efficiency ($\epsilon _\textrm{Ps} = 40$%) and laser excitation efficiency ($\epsilon _\textrm{ex} = 25$%). The right axis shows the number of Debye lengths per L=1 cm

Figure 10 shows the dependence of the Debye length on the lepton density, $n_e$, for mean energies of $\frac{3}{2} k_B T = 1$ eV (dotted line), 100 meV (solid line), and 10 meV (dashed line). The top axis indicates the estimated number of positrons required to achieve a certain number density, assuming $n_e / N_{e^+} = 10$ $\textrm{m}^{-3}$. This is consistent with a Ps production efficiency of $\epsilon _\textrm{Ps} = 40$%, laser excitation efficiency of $\epsilon _\textrm{ex} = 25$%, and a normalized lepton density of $n_e / N_{\textrm{Ps}^*} = 100$ $\textrm{m}^{-3}$. An initial bunch of $N \sim 4 \times 10^9$ $e^+$ would be needed to trap a 100 meV collection of electrons and positrons with a Debye length of $\lambda _D \approx 1$ cm. If the electrons and positrons cyclotron cool to T≈77 K, the Debye length would reduce to $\lambda _D \approx 3$ mm.

## Conclusions

Rydberg positronium injection is a conceptually simple technique for depositing bunches of low-energy electrons and positrons into an LD trap. In this work, the feasibility of implementing an RPI scheme was examined. Considerations include the production and laser excitation of Ps and the geometric acceptance of the APEX-LD trap. The critical components of the proposed RPI scheme have all been demonstrated experimentally [41, 50, 58].

Many phenomena relevant to RPI merit further consideration, including Rydberg–Stark deceleration [65, 84], Rydberg–Rydberg interactions [98, 99], Rydberg-plasma collisions [100, 101], magnetobound states [94], magnetized positronium [44, 45], and $e^+$/$e^-$ recombination [13, 102]. RPI is very tunable and annihilation γ-rays can be efficiently detected with excellent timing and energy resolution, allowing these effects to be investigated experimentally with exquisite control and powerful diagnostics [36, 103]. Understanding the nuances of collisions between magnetized particles is crucial for modeling transport in magnetically confined plasmas [104]. Magnetic confinement of equal numbers of electrons and positrons would provide a novel opportunity to investigate low-energy interactions between oppositely charged, equal-mass particles. This might yield important insights into the transport and annihilation of galactic positrons [105], even before plasma densities are attainable. RPI could also be used to embed a small number of positrons into an ion-electron plasma, which could facilitate the fusion-relevant transport studies originally envisioned by *Surko et al.* [43].

RPI cannot compete with drift injection in terms of efficiency [34]. However, it can produce overlapping, low-temperature distributions of electrons and positrons deep inside the APEX-LD confinement volume. The MC simulations suggest that a marginal pair plasma can be created using currently available $e^+$ trapping technology [21]. If the confinement time is long enough for cyclotron cooling, a plasma with a mm-scale Debye length is conceivable.

The geometry of APEX-LD is serendipitously well-suited to RPI—although it would be much easier to obtain plasma densities in a smaller version of the device. Extensions of the basic RPI concept could facilitate even higher injection efficiencies. For instance, instead of relying on the MSE, Rydberg Ps could be ionized by an electric field applied between the floating coil and a coaxial drift tube. Thin mesostructured materials that efficiently produce Ps in transmission [106] may also allow RPI to be utilized in more complicated magnetic geometries [13]. If a cm-scale magnetic trap could be built, photoionization of Ps would allow for precise control of the density and temperature of the electron and positron distributions [107].

## Data Availability

This manuscript has associated data in a data repository. [Authors’ comment: The Open Research Data Repository of the Max Planck Society https://doi.org/doi:10.17617/3.HORTKO.]

## Footnotes

1 High-*n* Ps atoms can experience Rydberg–Stark deceleration [65]; however, this effect is not included in the trajectory simulations.

2 The floating coil is assumed to be electrically neutral, even though it could acquire a charge. A large electric potential on the surface of the F-coil would result in the poloidal separation of the trapped $e^-$ and $e^+$ [73].
